# Human myogenic reserve cells are quiescent stem cells that contribute to muscle regeneration after intramuscular transplantation in immunodeficient mice

**DOI:** 10.1038/s41598-017-03703-y

**Published:** 2017-06-14

**Authors:** Thomas Laumonier, Flavien Bermont, Pierre Hoffmeyer, Vincent Kindler, Jacques Menetrey

**Affiliations:** 1 Department of Orthopedic Surgery, Geneva University Hospitals & Faculty of Medicine, Geneva, Switzerland; 2 Department of Hematology, Geneva University Hospitals & Faculty of Medicine, Geneva, Switzerland

## Abstract

Satellite cells, localized within muscles *in vivo*, are Pax7^+^ muscle stem cells supporting skeletal muscle growth and regeneration. Unfortunately, their amplification *in vitro*, required for their therapeutic use, is associated with reduced regenerative potential. In the present study, we investigated if human myogenic reserve cells (MRC) obtained *in vitro*, represented a reliable cell source for muscle repair. For this purpose, primary human myoblasts were freshly isolated and expanded. After 2 days of differentiation, 62 ± 2.9% of the nuclei were localized in myotubes and 38 ± 2.9% in the mononucleated non-fusing MRC. Eighty percent of freshly isolated human MRC expressed a phenotype similar to human quiescent satellite cells (CD56^+^/Pax7^+^/MyoD^−^/Ki67^−^ cells). Fourteen days and 21 days after cell transplantation in immunodeficient mice, live human cells were significantly more numerous and the percentage of Pax7^+^/human lamin A/C^+^ cells was 2 fold higher in muscles of animals injected with MRC compared to those injected with human myoblasts, despite that percentage of spectrin^+^ and lamin A/C^+^ human fibers in both groups MRC were similar. Taken together, these data provide evidence that MRC generated *in vitro* represent a promising source of cells for improving regeneration of injured skeletal muscles.

## Introduction

Satellite cells (SC) are muscle stem cells located between the plasma membrane of the muscle fibers and the surrounding basal lamina and are essential for muscle regeneration^1, 2^. In uninjured muscle, SC are mostly quiescent. In response to mechanical stress or injury, SC become activated, proliferate and undergo either differentiation to form new myofibers or self-renew to replenish the SC pool. These diverging fates are associated with variation in Pax7 and MyoD expression pattern: Pax7^+^MyoD^−^ (quiescent SC), Pax7^+^MyoD^+^ (activated SC or proliferating myoblasts) and Pax7^−^MyoD^+^ (differentiating cells)^3, 4^. Myoblasts derived from SC activated *in vitro* are readily expandable and are considered as a potential tool for cell-based therapies aimed at the regeneration of skeletal muscle. So far, and despite encouraging studies in *mdx* mice^5, 6^, clinical trials, involving the injection of human allogeneic myoblasts in dystrophic patient muscles, have not been fully successful^7–9^. These observations indeed revealed the limited life span, migration, and/or proliferation capacities of grafted allogenic human myoblasts *in vivo* ^10, 11^, which are most probably induced by inappropriate culture conditions prior to injection. Other muscle stem cells sources were investigated including mesoangioblasts/pericytes^12–14^, induced pluripotent stem cells^15^, and CD133^+^ myogenic cells^16–18^, all demonstrating a high myogenic potential *in vitro* and *in vivo*. Among these, the impact of intra-arterial injection of human mesoangioblasts to restore dystrophin expression in Duchenne patients was recently evaluated. The process was found safe but its clinical efficacy very limited^19^.

SC are still considered as a potent source for muscle regeneration therapies^11, 20, 21^ but the difficulty to expand them *in vitro* without spoiling their regenerative potential remains^22, 23^. One possible alternative to SC is the yogenic reserve cells (MRC), that are quiescent myogenic cells, appearing during *in vitro* culture of myoblasts^24–26^, and whose regenerative characteristics deserves further investigation.

In this study, we generated and characterized human MRC and assessed their potential as a source of myogenic stem cells able to improve muscle regeneration *in vivo*. For this purpose, we generated high amounts of human MRC from freshly isolated primary human myoblast cultures and transplanted them in immunodeficient mice. We demonstrated that 80% of human MRC were quiescent Pax7^+^/MyoD^−^ cells, and importantly, that human MRC exhibited an enhanced survival and an enhanced potency to generate Pax7^+^ cells after transplantation in immunodeficient mice compared to human myoblasts. These data highlight a potential role of human MRC in improving muscle healing and in the maintenance of a pool of SC *in vivo*.

## Results

### Generation of human myogenic reserve cells

**Figure 1:**
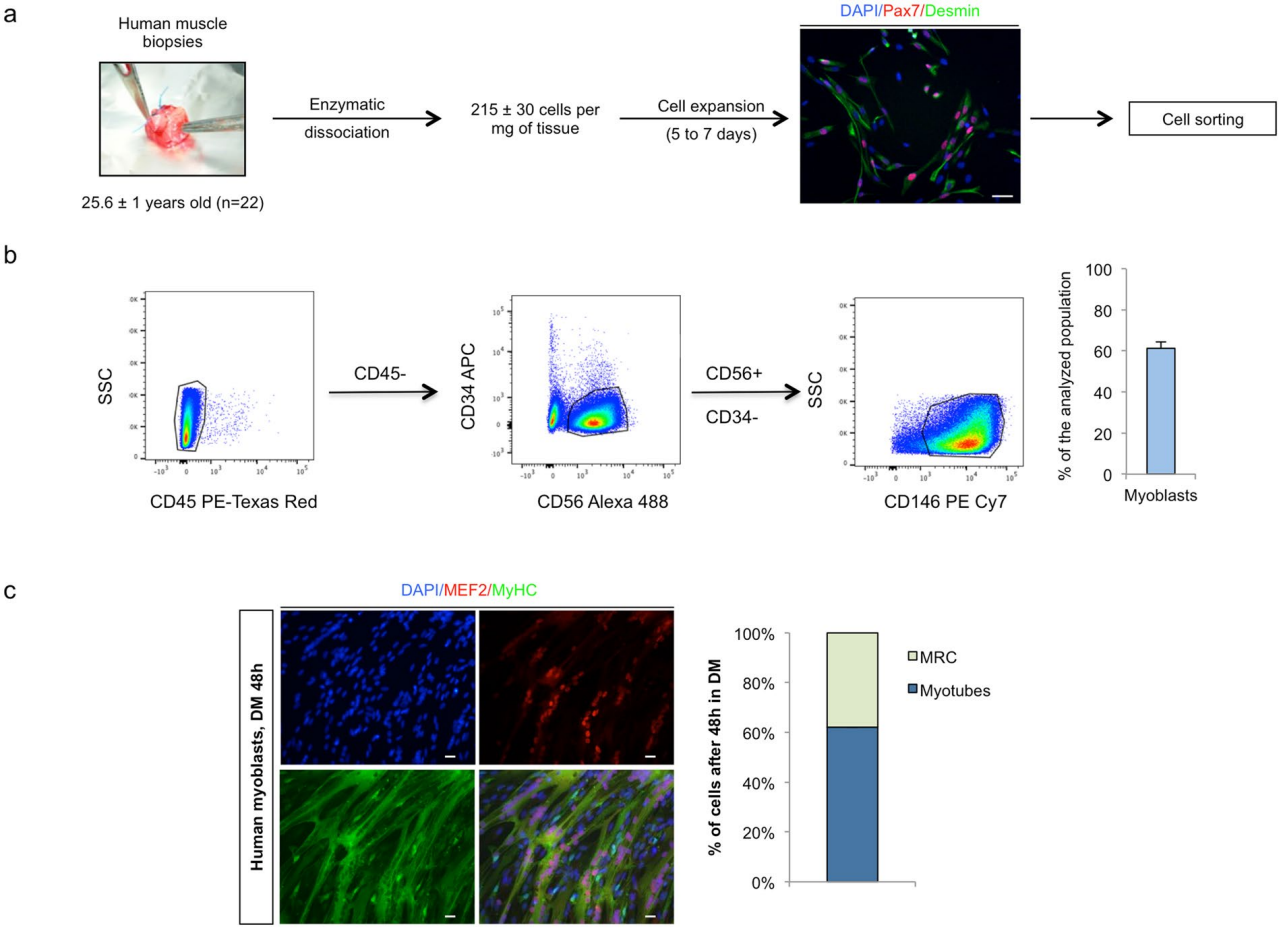
Generation of myogenic reserve cells. (**a**) Scheme of myoblast extraction and amplification before sorting. (**b**) Strategy of myoblast sorting by flow cytometry and purity determination post sort. CD45^+^ cells were excluded by sorting and the remaining CD45^−^ population was analyzed based on CD56, CD144 (not shown) and CD34 expression. CD56^+^/CD144^−^/CD34- populations were further gated on CD146 to identify human myoblasts (defined as CD56^+^/CD146^+^/CD45^−^/CD144^−^/CD34^−^ cells). These represented 61.2 ± 3.1% of the sorted population (mean ± SEM, n = 22). (**c**) Sorted cells were further amplified, exposed to 48 h differentiation medium (DM), fixed and stained with antibodies against MEF2 (red), Myosin Heavy Chain (MyHC, green) and with DAPI (blue) to identify nuclei. Myotubes defined as DAPI+/MEF2+/MyHC+ cells and myogenic reserve cells (MRC) defined as DAPI+/MEF2−/MyHC− cells represent respectively 62 ± 2.9% and 38 ± 2.9% (mean ± SEM, n = 6) of the myogenic population. Images shown are representative of 6 independent experiments. Scale bars: 20 μm.

After enzymatic dissociation of human muscle biopsies (individuals of 25.6 ± 1 years old, n = 22, 215 ± 30 cells per mg of muscle tissue), cells were amplified for 5 to 7 days in growth medium (GM). CD56^+^/CD146^+^/CD45^−^/CD34^−^/CD144^−^ human myoblasts were obtained after flow cytometry cell sorting. Myoblasts represented 61.2 ± 3.1% of the analyzed population (Fig. 1a and b).

Myoblasts were replated, grown to confluence and cultured in differentiation medium (DM) for 48 hours. A majority of the cells formed myotubes *in vitro* (MF20^+^/MEF2^+^ cells) with fusion index values of 62.0 ± 2.9% and 38.0 ± 2.9% of cells were human myogenic reserve cells (MRC), escaping the terminal differentiation process (Fig. 1c).

### Characterization of human MRC obtained after 48 hours of differentiation *in vitro*

**Figure 2:**
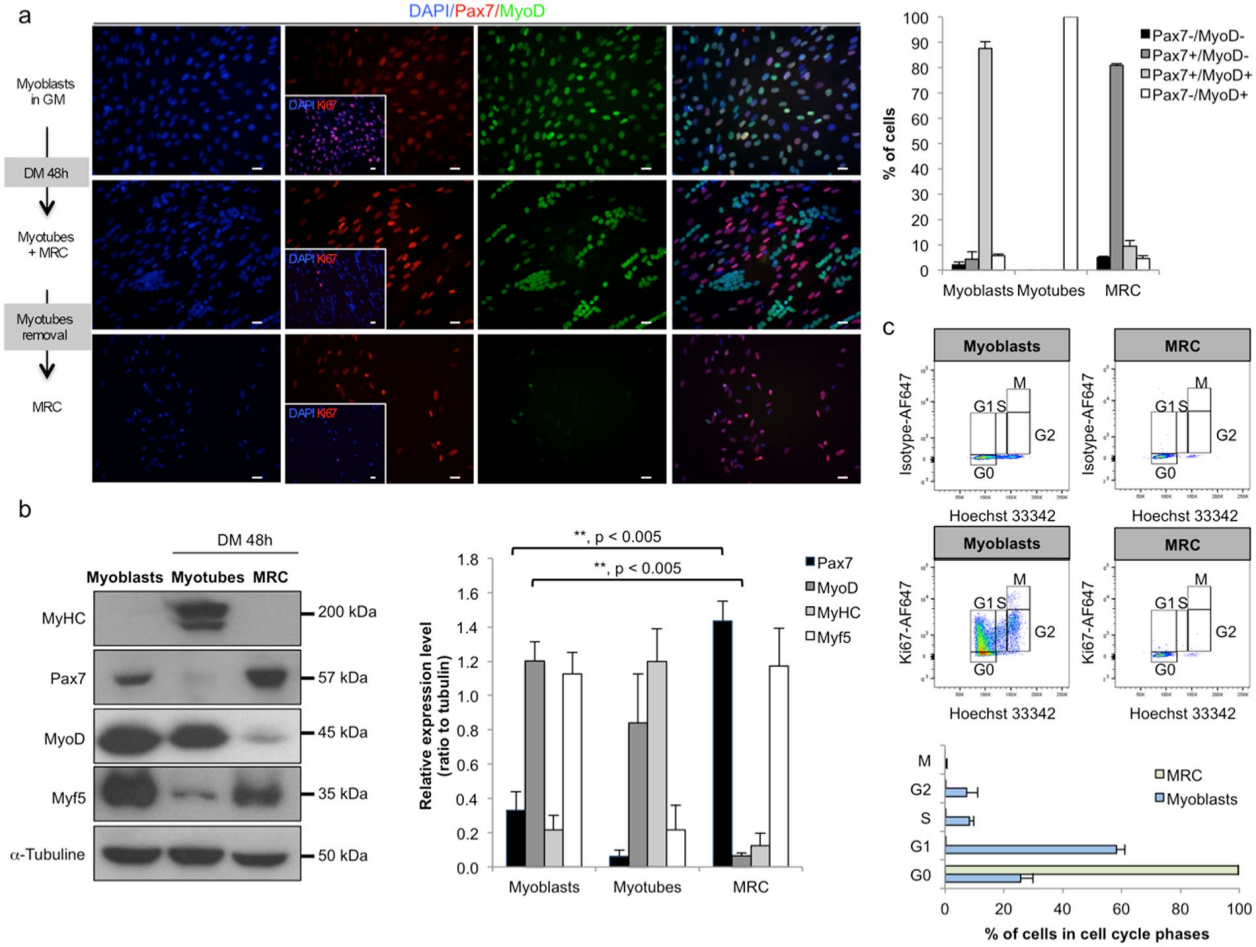
80% of human MRC are quiescent myogenic Pax7^+^/MyoD^−^ cells. (**a**) Immunofluorescence of human myoblasts cultures exposed to GM or to DM 48 h, fixed and stained with antibodies against Pax7 (red), MyoD (green) and DAPI (blue). Inserts display Ki67 staining (red). The proportion of Pax7^+/−^/MyoD^+/−^ cells in myoblasts, myotubes or MRC population, as established from the slide are represented in graph (mean ± SEM, n = 3). (**b**) Western blot analysis of human myoblasts, human myotubes and human MRC. Data are representative of 4 independent experiments for western blots and the graph plotted is the mean ± SEM of 4 independent experiments. Scale bars: 20 μm. (**c**) Cell cycle analysis of human myogenic cells determined by FACS. After trypsinisation, myoblasts and MRC were fixed, permeabilized and stained with Hoechst 33342 and anti-Ki67 mAb conjugated to AlexaFluor®647. Left upper panels IgG1κ AlexaFluor®647-labeled isotype control. Left lower panels: specific antibody labeling; the gates of G0/G1/S/G2/M for each population (human myoblasts or human MRC) are displayed. Right histogram: data shown are mean ± SEM of 3 independent experiments derived from flow cytometry analysis.

**Figure 3:**
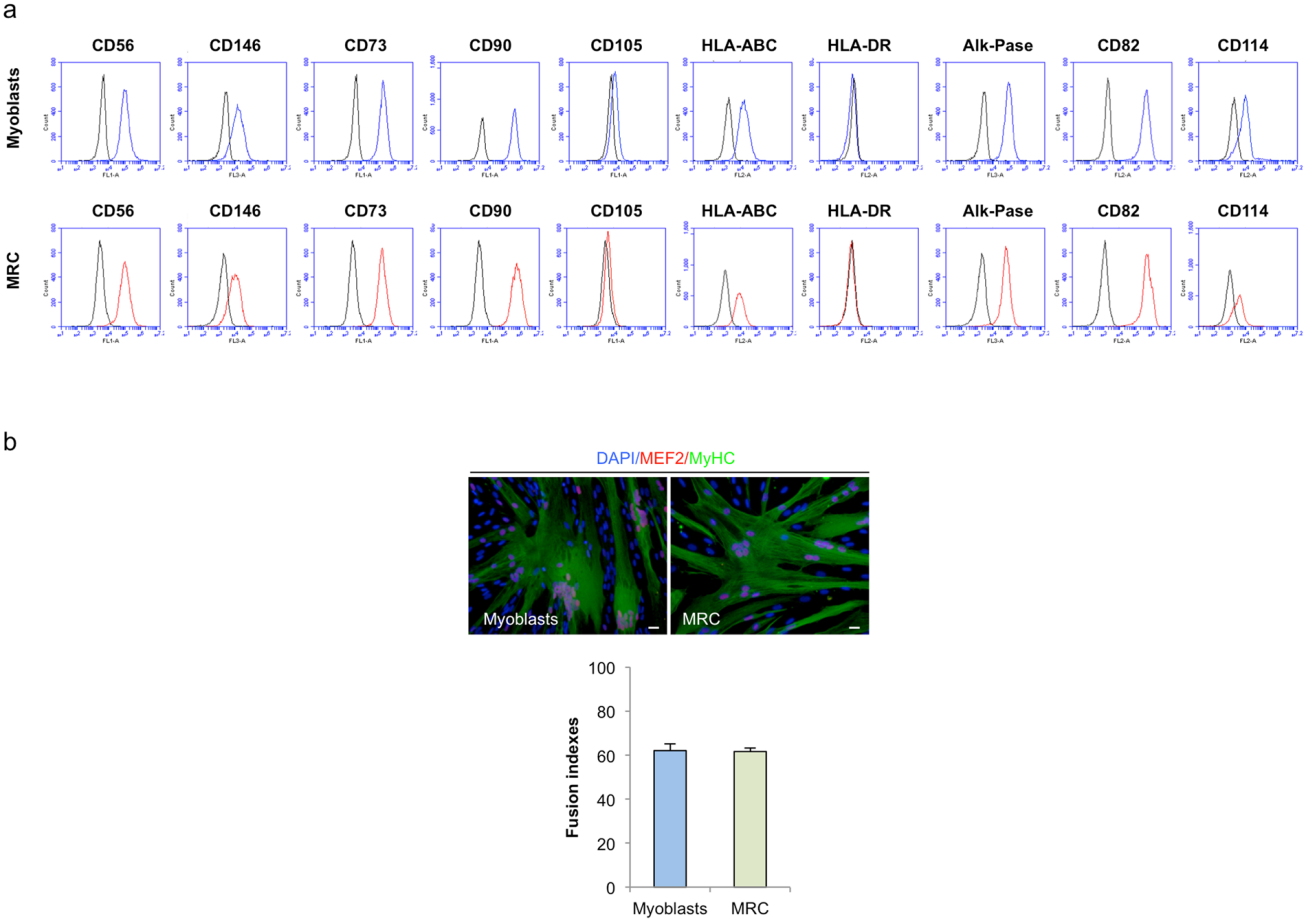
Characterization of human myoblasts and human MRC by flow cytometry analysis. (**a**) Human myoblasts and human MRC (respectively blue and red curve), were analyzed for various cell surface markers. Isotypic controls are shown in black. Data shown are representative of 6 independent experiments. (**b**) MRC fused in DM after growth in GM, with fusion indexes similar to human myoblasts (61.62 ± 1.6% and 62 ± 2.9% respectively, mean ± SEM, n = 6, p > 0.05). Scale bars: 20 µm.

We analyzed, by immunofluorescence, the level of Ki67, Pax7 and MyoD expression in proliferation- and differentiation-inducing cultures.. In proliferation (GM), the majority of human myoblasts was Ki67^+^ (81.9 ± 1.5% of Ki67^+^ cells, mean ± SEM, n = 3) and Pax7^+^/MyoD^+^ (87.7 ± 2.6%, n = 3, Fig. 2a). After 2 days in DM, all cells were Ki67^−^. The main population consisted in Pax7^−^/MyoD^+^ post-mitotic cells forming myotubes (Fig. 2a). The remaining population consisted in non-fused mononucleated cells, defined as MRC. MRC were composed of 80.8 ± 0.7% Pax7^+^/MyoD^−^ cells, 9.4 ± 2.3% of Pax7^+^/MyoD^+^, 4.7 ± 0.9% of Pax7^−^/MyoD^+^ and 5 ± 0.4% of Pax7^−^/MyoD^−^ (n = 3 for all, see Fig. 2a). MRC and myoblasts cell cycle were further analyzed by flow cytometry after a Ki67 and Hoechst 33342 staining. As expected from Fig. 2a, MRC were quiescents, with 99.6% of the population in the G0 phase and few Ki67^−^ cells locked in S/G2 (n = 3, Fig. 2c) while only 25.8% of myoblasts were in G0, 58.2% in G1 and 16% within the S-G2-M gates (n = 3, Fig. 2c). Various cell surface markers such as CD56, CD146 CD73, CD82, CD90, CD105, CD114, alkaline phosphatase and HLA-ABC were analyzed on both myoblasts and MRC, and were found to be expressed at similar levels (Fig. 3a).

The expression pattern of myogenic factors (Pax7, MyoD, MyHC and Myf5) was also analyzed in myoblasts, myotubes and MRC by Western blot (n = 4). Proliferating myoblasts (in GM) expressed high level of MyoD and low level of Pax7. In MRC, compared to myoblasts, Pax7 expression was enhanced by 4 fold whereas MyoD expression was massively down regulated to reach very low levels (P < 0.005, n = 4, Fig. 2b). Myf5 expression was similar in myoblasts and in MRC but down regulated in myotubes. Purity of our cell preparation was confirmed by myosin heavy chain (MyHC) that is expressed only in myotubes extracts.

Finally, the myogenic potential of MRC generated *in vitro* was tested. Myotubes were removed from the culture dishes and exposed the remaining adherent mononuclear MRC to GM. This induced cell proliferation until confluence and upon exposure to DM, cell fusion ensued, reaching a level comparable to that observed in initial culture of human myoblasts (fusion index of 61.62 ± 1.6%; Fig. 3b, n = 6). This result confirmed that MRC are myogenic cells.

### The proportion of Pax7^+^/MyoD^−^ cells in MRC population diminished with time in differentiation

**Figure 4:**
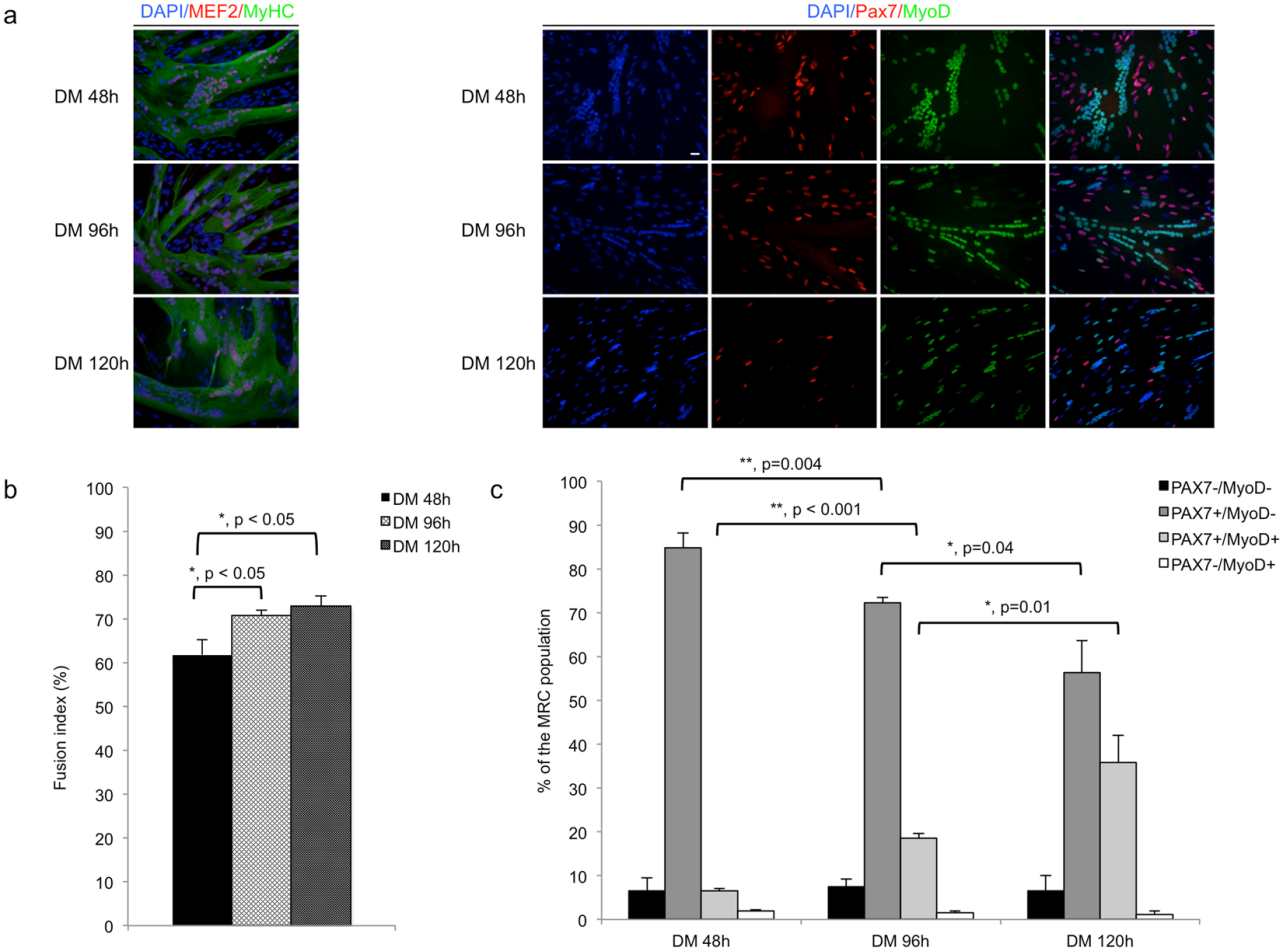
The proportion of Pax7^+^/MyoD^−^ cells in MRC population decreases with time in differentiation. (**a**) Cultures of human myoblasts exposed to DM 48 h, DM 96 h and DM 120 h, were fixed and stained with anti-MEF2 (red) anti-MyHC (green) or anti-Pax7 (red)/MyoD (green) mAb, and with DAPI (blue). Images shown are representative of 3 independent experiments. Scale bars: 20 μm. (**b**) Fusion indexes (number of nuclei counted in MyHC positive myotubes) were calculated 48 h, 96 h and 120 h after differentiation initiation. Fusion indexes were significantly increased at 96 h and 120 h as compared to 48 h with value of 70.8 ± 2%, 73 ± 2.3% and of 61.9 ± 2.2% respectively (mean ± SEM, n = 3; P < 0.05). (**c**) The proportion of Pax7^+^/MyoD^−^ cells was significantly smaller, and that of Pax7^+^/MyoD^+^ cells significantly higher after 96 h and 120 h of incubation in DM compared to 48 h incubation (p < 0.05 using unpaired Student’s t test with Bonferroni correction). Data shown bar charts are mean ± SEM of 3 independent experiments.

We computed fusion indexes and characterized the MRC population 48 h, 96 h and 120 h after differentiation initiation. Fusion indexes averaged 61.9 ± 2.2% after 48 h in DM and increased to 70.8 ± 2% and 73 ± 2.3% respectively after 96 h and 120 h in DM (n = 3; P < 0.05, Fig. 4a). Moreover, the proportion of Pax7^+^/MyoD^−^ cells in MRC population decreased with increasing exposure to DM, reaching 84.9 ± 3.4%, 72.3 ± 1.2% and 56.4 ± 7.3% respectively after 48 h, 96 h and 120 h in DM (n = 3, P < 0.05). By contrast, the proportion of Pax7^+^/MyoD^+^ cells in MRC population significantly increased with increased exposure to DM, reaching 6.5 ± 0.6%, 18.6 ± 1.1% and 35.8 ± 6.2% after 48 h, 96 h and 120 h in DM, respectively (n = 3, P < 0.05, Fig. 4b).

### Improved survival of MRC as compared to human myoblasts after injection in lacerated murine muscles

**Figure 5:**
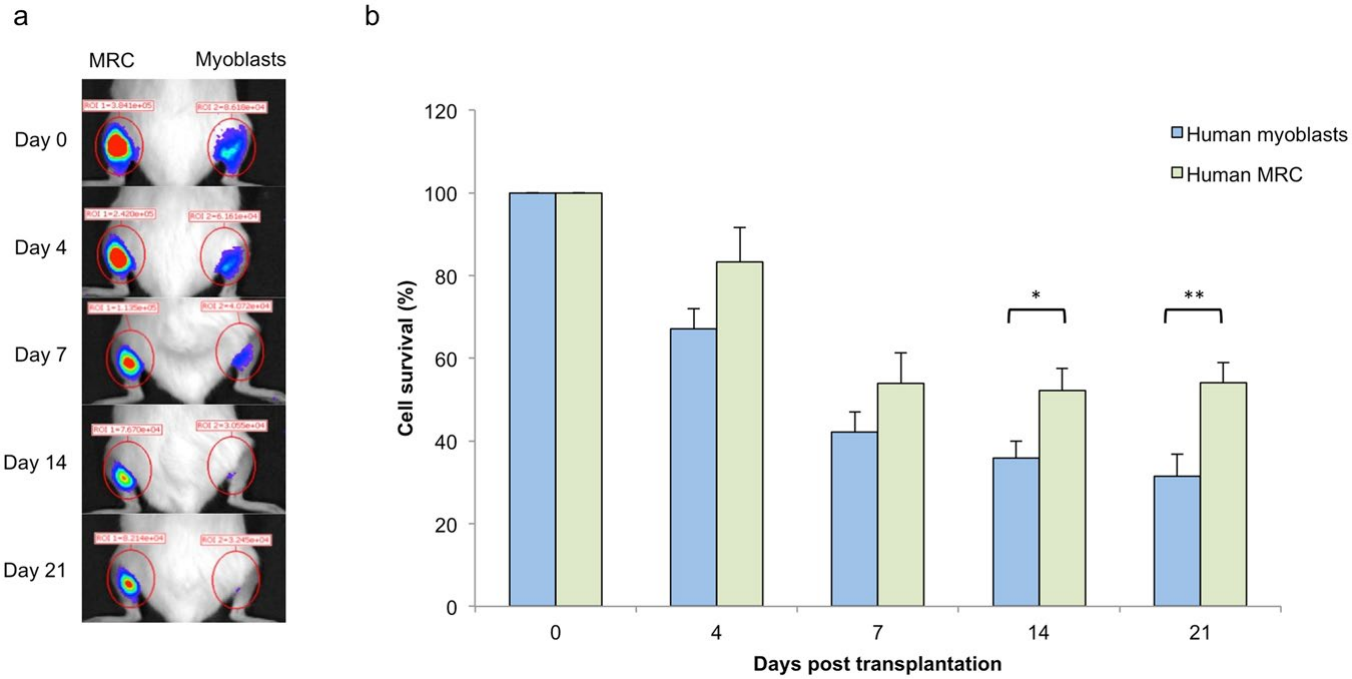
Enhancement of human MRC survival as compared to human myoblasts after xenotransplantation in lacerated murine muscles. (**a**) Representative bioluminescence images of a time course analysis of one mouse transplanted with either 5 × 10^5^ MRC or 5 × 10^5^ myoblasts, quantified in (**b**). The percentage of surviving cells was normalized respective to the signal measured 3 h after cell injection (day 0), and considered as 100% in each group. Data are represented as mean ± SEM (n = 12; *p < 0.05; **p < 0.001, using unpaired Student’s t test).

Human quiescent Rluc^+^ and proliferating myoblasts Rluc^+^ were injected in lacerated Gastrocnemius muscles of immunodeficient mice and cell survival was quantified by bioluminescence imaging (BLI) at various time point. The percentage of cell survival at day 4, 7 14 and 21 always refer to the 100% survival obtained by measuring BLI 3 h after cell transplantation for each cell injection. No significant difference in the percentage of human live cells remaining in mice was observed at day 4 and day 7 between the 2 groups. By contrast, differences in human cell survival were observed 14 days after cell injection (52.3 ± 4.1% vs. 35.9 ± 5.2% for MRC and myoblast respectively) and 21 days after cell injection (54.1 ± 5.2% vs. 31.5 ± 4.8% for MRC and myoblast respectively). These differences were statically significant (P < 0.05, n = 12, Fig. 5a,b), suggesting that MRC survived better after xenotransplantation than that of myoblasts.

### MRC and myoblasts contribute equally to lacerated muscle regeneration

**Figure 6:**
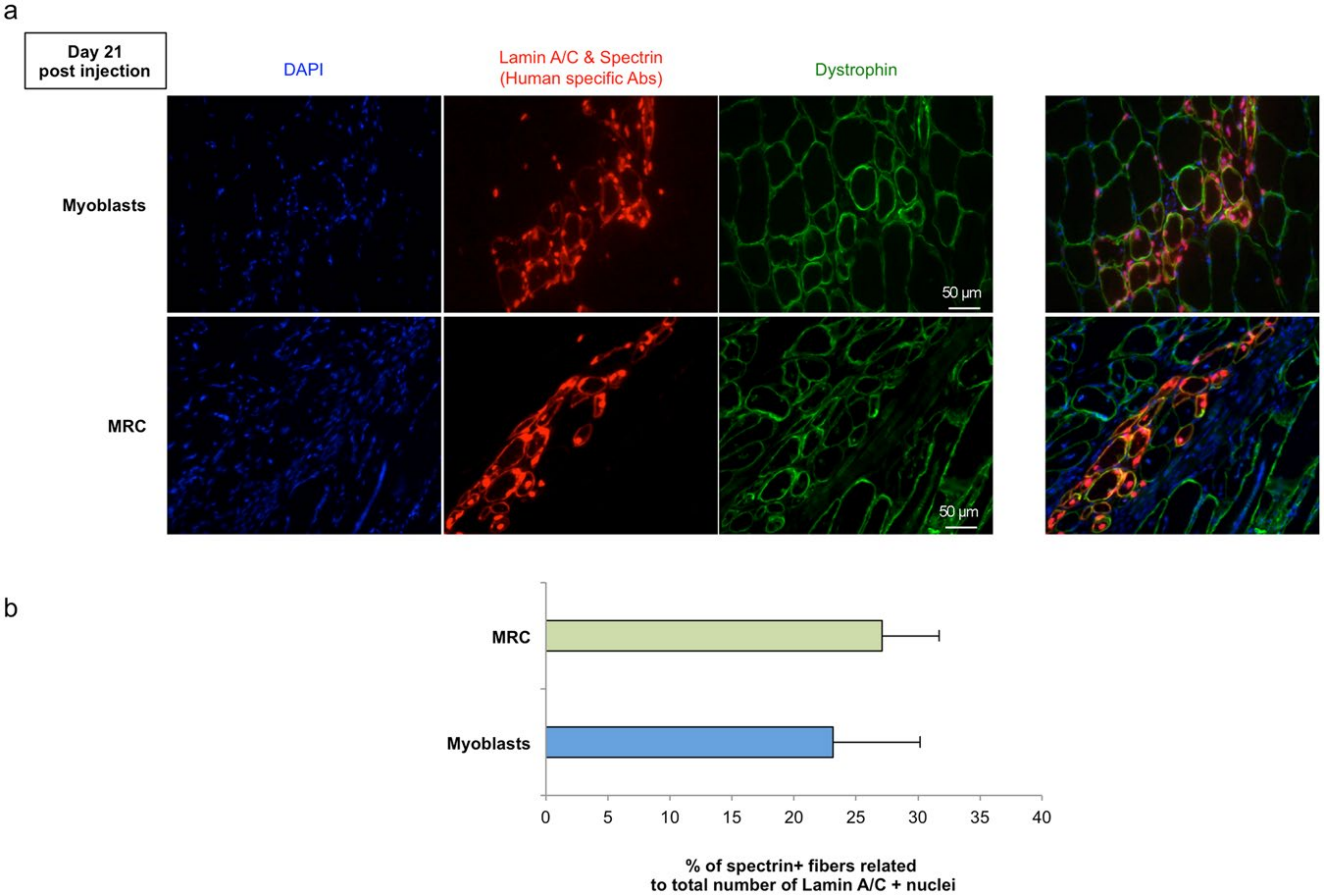
Engraftment assessment 21 days after intramuscular injection of human myogenic cells in lacerated *Gastrocnemius* muscles of immunodeficient mice. (**a**) Immunofluorescent staining with anti dystrophin antibody (green), anti-human spectrin anti-human lamin A/C mAb (red fibers and red nucleus) and DAPI (blue), identifies hybrid myofibers incorporating either human myoblasts or human MRC. Scale bar: 50 μm. (**b**) Means values are show on the lower plot (mean ± SEM, n = 6).

Skeletal muscle fibers of human origin were defined as fibers containing two human-specific markers, i.e., human spectrin and a human lamin A/C. We also used an antibody against mouse/human dystrophin to reveal muscle fibers of both human and murine origin. Quantification of human fibers was defined as the ratio of spectrin positive fibers to total number of Lamin A/C positive nuclei to compensate for variation in the number of human cells present in an injected muscle section. Three weeks after cell injection, transplanted myoblasts and MRC contributed equally to muscle regeneration as indicated by the presence of a similar percentage of spectrin^+^ human fibers (related to the total number of lamin A/C^+^ cells) in both groups (23.1 ± 7% and 27.1 ± 4.6% respectively for myoblasts and MRC; P = 0.6, n = 6, Fig. 6).

### Pax7^+^ cells of human origin are more numerous after MRC injection than myoblast injection

**Figure 7:**
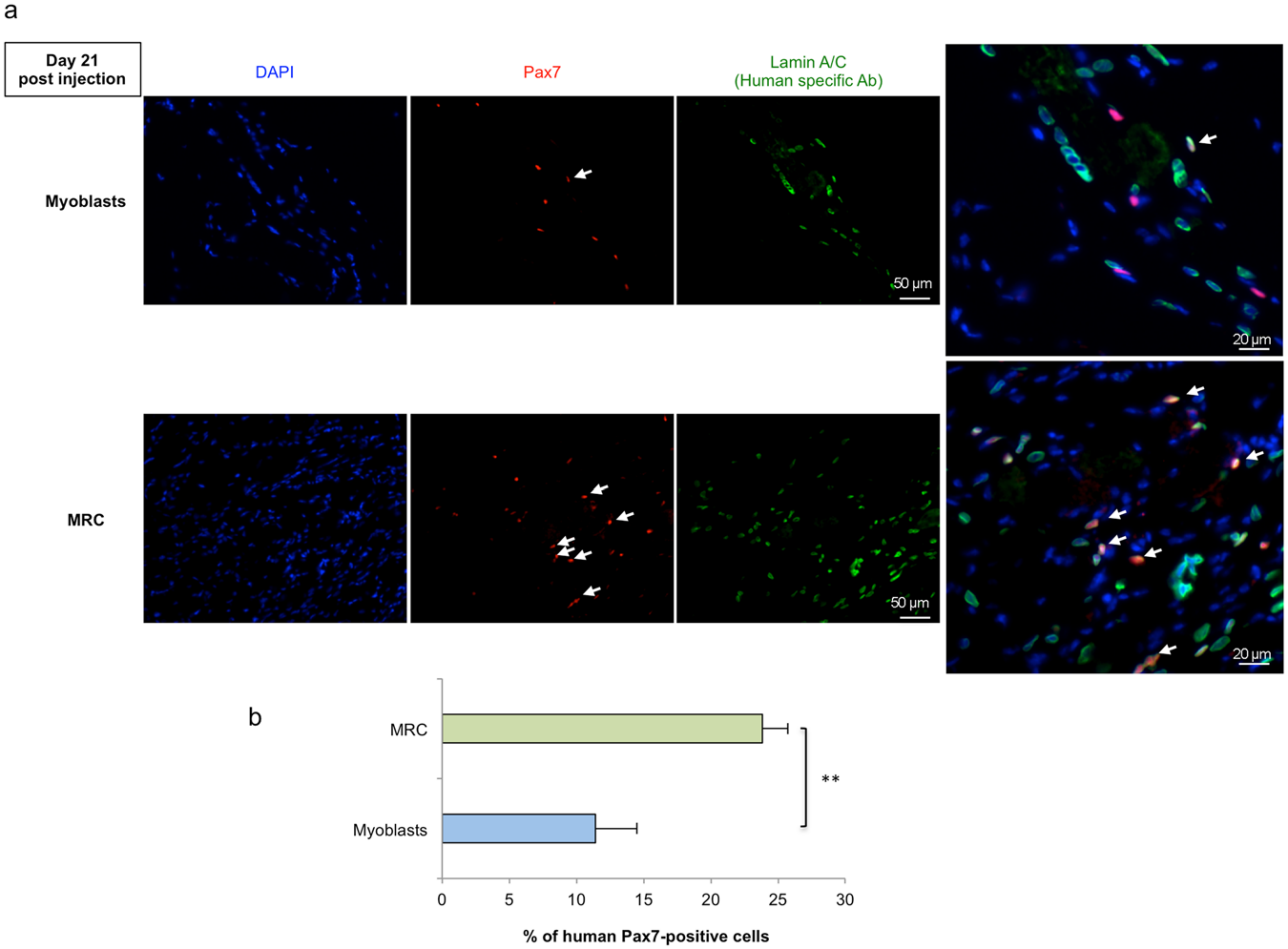
Pax7^+^ myogenic cells detected 21 days after intramuscular are more numerous after injection of MRC compared to myoblasts. (**a**) Twenty-one days after myoblasts or of MRC intramuscular injection, muscle sections were double stained with an anti-human lamin A/C (green) and anti-Pax7 (red). Nuclei were counterstained with DAPI (blue). Lamin A/C^+^ and Pax7^+^ positive cells (white arrow) were observed within murine muscle injected either with human myoblasts or with human MRC. Scale bar: 50 μm. (**b**) Mean percentage ± SEM of human Pax7^+^ cells (number of Pax7^+^ cells/total number of LaminA/C^+^ cells) in representative sections of muscle injected with MRC or myoblasts are shown (n = 6; **p < 0.001, using unpaired Student’s t test).

**Figure 8:**
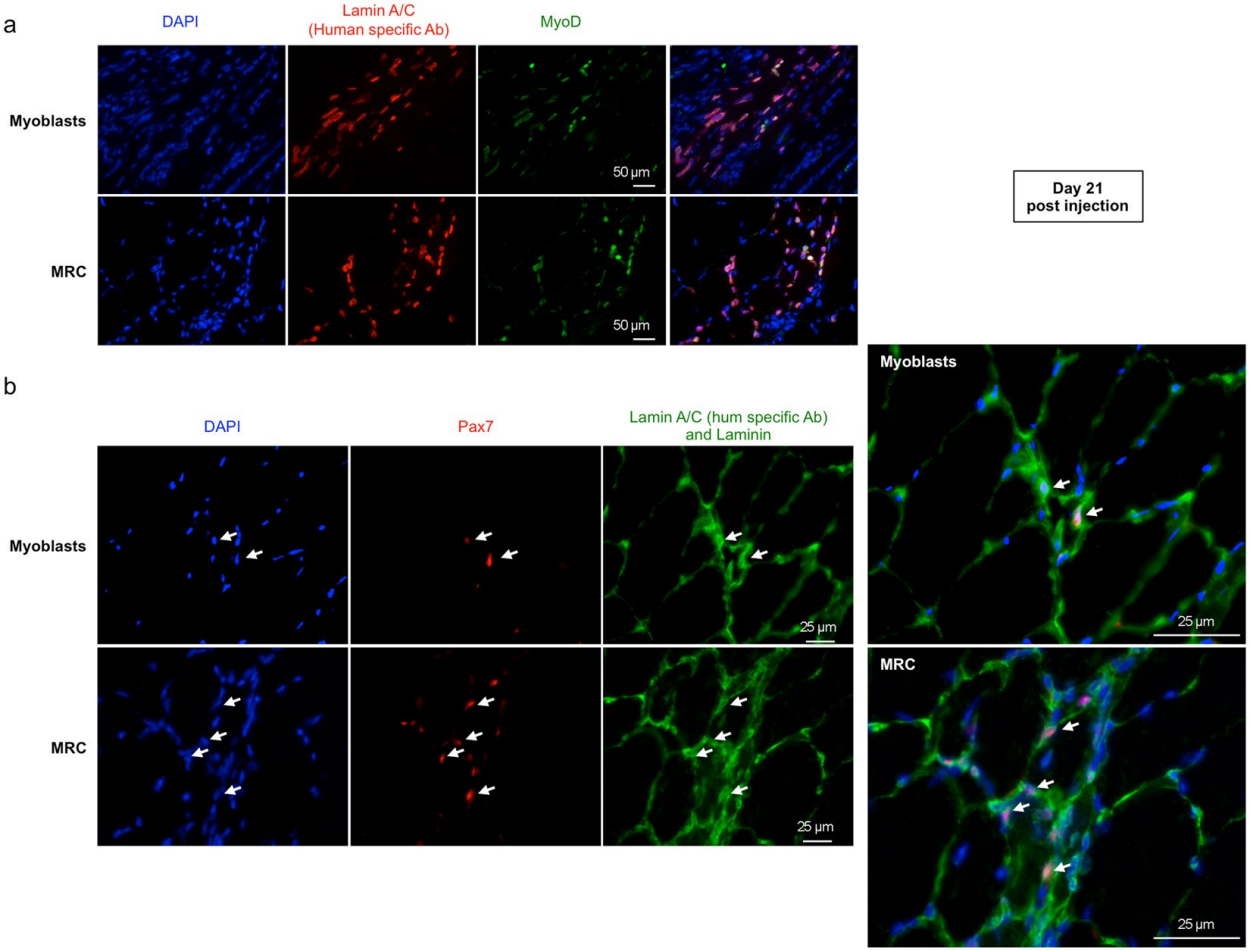
MyoD expression and *in vivo* localization of human myoblasts and of human MRC after transplantation. Twenty-one days after human myoblasts or MRC intramuscular injection, muscle sections were stained (**a**) with anti-human lamin A/C (red) and anti-MyoD (green) mAb. In panel (**b**), muscle sections were stained with anti-human lamin A/C (green nuclei), anti-laminin (green) and anti-Pax7 (red). Nuclei were counterstained with DAPI (blue). Some human lamin A/C^+^Pax7^+^ myogenic cells (white arrow) were observed beneath the basal lamina protein, laminin.

To identify SC of human origin in grafted mice, muscle sections from animals injected with either myoblasts or MRC, were stained with antibodies against human lamin A/C and the SC marker Pax7 (Fig. 7). This showed that the percentage of human lamin A/C^+^/Pax7^+^ cells (indicated by white arrows) in representative muscle sections was significantly increased after MRC injection compared to myoblast injection (23.8 ± 1.9% vs. 10.1 ± 1.7% of lamin A/C^+^Pax7^+^ cell respectively (n = 6, Fig. 7). We further observed that injected human myoblasts and human MRC express MyoD at 21 days after injection (Fig. 8a). *In vivo* localization of these cells was also investigated using an antibody directed against laminin, a basal lamina protein that cover the SC niche. Double positive cells for Pax7 and human Lamin A/C were localized surrounded by laminin suggesting that some human cells adopt a SC position after intramuscular injection (Fig. 8b).

## Discussion

Myogenic precursor cell transplantation is one option for the treatment of Duchenne patients and/or for the treatment of skeletal muscle injuries^21, 27^. Satellite cells (SC) and their progeny, myoblasts, are essential for muscle regeneration to occur^2^ and as such, may represent an “ideal” cell source to restore skeletal muscle integrity. Unfortunately, the *in vitro* amplification process required to obtain therapeutic doses of myoblasts, reduces their regenerative capabilities^23, 28^. Myogenic reserve cells (MRC) have been identified 20 years ago *in vitro*, as cells able to escape spontaneously terminal differentiation but which remain myogenic if further stimulated^24^. In the present study, we demonstrated that human MRC are similar to quiescent human SC, participate to muscle regeneration with an enhanced potency compared to myoblasts to generate Pax7^+^ cells *in vivo*, and apparently fulfill all the criteria expected from a therapeutic tool.

SC fate is governed by the expression of various transcription factors including Pax7 and MyoD. Pax7 is expressed in quiescent and activated satellite cells, whereas MyoD is expressed in activated satellite cells and during differentiation^29, 30^. In our *in vitro* model, we generated a heterogeneous population of MRC after differentiation initiation. A majority of these cells (80%) were Pax7^+^/MyoD^−^/Ki67^−^ cells, similar to quiescent SC. Down-regulation of MyoD, which is known to be required for generation of MRC^26, 31^, was confirmed by western blot analysis. A number of properties of MRC described *in vitro* are similar to those of quiescent muscle SC. Likewise, 80% of human MRC are quiescent mononucleated Pax7^+^/MyoD^−^ cells, and proliferate in GM while retaining a high differentiation potential in DM. Two markers, CD82 and CD114 (G-CSF receptor), recently identified in myogenic precursor cell populations^32, 33^ were also expressed at similar level by human MPC and human MRC.

Compared to earlier studies of damaged muscles regeneration by xenogenic myoblasts^34–38^, we obtained in this study a mean survival of human myoblasts 7 days after implantation which was 4 fold superior compared to that of grafted clonal human myoblasts described in another study^38^. This could be explained in part by the use of severe immunodeficient NOD/Shi-scid, IL-2R γ null (NOG) mice^39^, that lack mature lymphocytes (B, T), natural killer cells, have dysfunctional macrophages and dendritic cells, and a reduced complement activity. Such altered dynamics of the early innate response and immune cellular reactions may favor early myoblasts survival post injection^40^. Moreover, the *in vitro* cell expansion protocol we used which involved polyclonal myoblasts rather than clonal myoblasts decreases the number of cell doublings prior to injection and may favor survival^23, 41^. Finally, the state of the muscle at the time of injection may also affect its regeneration^42, 43^, such as the laceration of the muscle prior to injection may favor xenogenic cell survival.

An important finding of the present study is the difference in cell cycle status between MRC and myoblasts at the end of the *in vitro* culture immediately prior to transplantation, and the significant difference in survival/engraftment in the host between these cells observed 14 and 21 days after transplantation. The increased engraftment seems not to be due to a differential expression of myogenic markers such as CD56, CD82 or CD146 or the myogenic regulatory factor Myf5, since these were expressed at a similar level on both cell types. By contrast MyoD expression may impact on graft take since it has been reported that myogenic cells lacking or with reduced MyoD expression, implant more efficiently than cells expressing high level of MyoD^44, 45^. It may thus be expected that myoblasts which express MyoD would exhibit a reduced ability to engraft *in vivo* ^16, 23, 46^, while MRC whose 80% do not express MyoD by the time of grafting (if undertaken after 48 hours of exposure to DM, see our data) may engraft with a higher efficiency than myoblasts early after transplantation. Nevertheless, as already demonstrated for human myoblasts that were shown to differentiate rapidly after implantation *in vivo* ^47^, we also observed that myoblasts and MRC express MyoD at 21 days after injection.

The difference in cell cycle observed between the 2 cell types at the time of injection may also impact grafting efficiency. Quiescent MRC may adopt a metabolic profile favorable to their survival and increase their autophagy as demonstrated recently for quiescent preconditioned mesenchymal stem cell^48^. As such, initial quiescence may favor myogenic stem cell survival after transplantation in an ischemic environment. This in accordance with a recent study showing that satellite cells maintained in an undifferentiated stage by leukemia inhibitory factor engrafted more efficiently^49^. The mechanisms involved in the regulation of muscle stem cell quiescence are not fully identified yet, but mechanisms involving Sprouty1^50, 51^, autophagy^52^, notch signaling^53^, miRNAs^54^, Kruppel-like factor 7^55^ and epigenetic events^56^ could favor quiescent MRC emergence.

Another important property of human myogenic cells is their ability to regenerate injured muscle. MRC participated to muscle regeneration, as revealed by the presence of human nuclei in host myofibers positive for human spectrin. The ratio of Spectrin + fibers to total number of Lamin A/C + nuclei was rather low but similar to results obtained after human myoblast transplantation. Since these results could not explain the increased survival observed with MRC as compared to myoblasts, we hypothesized that MRC should better survive as non-fusing cells.

Using a Pax7 antibody, we demonstrated that human MRC gave rise to significantly more progenitor myogenic cells, as compared to human myoblasts. Thus, the fate of human myoblasts versus human MRC is different after intramuscular injections in a xenogenic host. A subpopulation (25%) of the injected quiescent MRC did not form myotubes although expressing Pax7. Some of these double positive Pax7/Lamin A/C cells adopt a satellite cell position. Their ability to respond to future muscle damage was not tested in our model but one may expect some of these cells to be at least in part functional, as observed after injection of myogenic CD133^+^ cells^16^ or human satellite cells^57^. In summary, we have demonstrated that human MRC are quiescent Pax7^+^/MyoD^−^ cells, with enhanced survival and enhanced potency to form Pax7^+^ cells *in vivo* as compared to that of human myoblasts. Compared to other potential myogenic stem cells^16, 19, 58^, MRC hold the advantage to be generated *in vitro* in number compatible with future therapeutic applications. It will be important to examine and compare the expression of genes involved in MRC and myoblasts self-renewal, to identify which pathway is differently activated between these two cell types. Their respective metabolic profiles should also be examined to decipher the possible metabolic shift existing between MRC and myoblasts. The use of artificial niche that maintain stem cell quiescence may also improved the therapeutic potential of MRC^59^.

## Methods

### Human muscle biopsies

Human muscle biopsies were collected during orthopedic surgery of 22 healthy patients (25.6 ± 1 years old). All methods relating to the human study were performed in accordance with the guidelines and regulations of the Swiss Regulatory Health Authorities and approved by the Comission Cantonale d’Ethique de la Recherche from the Geneva Cantonal Authorities, Switzerland (protocol CER n° 12–259). Informed and written consents were obtained from all subjects involved in the study.

### Mouse strain

All animal experiments were carried out according to the protocols approved by the ethics committee at the Cantonal Veterinary Office of Geneva, Switzerland (authorization N° 1048/3884/3). Female immunodeficient CIEA-NOG mice (10 to 16 weeks old) were purchased from Taconic (Ejby, Denmark) and were used in all *in vivo* experiments. Mice were maintained in the animal facility of the Geneva University Medical Center, Geneva, Switzerland and used in compliance with the local rules of animal experimentation approved by the Swiss Veterinary Office.

### Primary human myoblasts cell culture

Human skeletal muscles were minced and subjected to enzymatic dissociation (Trypsin EDTA 0,05%, Gibco) for 60 min under agitation at 37 °C. The process was stopped with 10% FCS. The cell suspension was then filtered through a 70 μm and a 40 μm nylon filter. Cell suspension was expanded in growth medium (GM) consisting of Ham’s F10 (Life Technologies) supplemented with 15% FCS (Life Technologies), bovine serum albumin (Sigma-Aldrich; 0.5 mg/ml), fetuin (Sigma-Aldrich; 0.5 mg/ml), epidermal growth factor (Life Technologies; 10 ng/ml), dexamethasone (Sigma-Aldrich; 0.39 μg/ml), insulin (Sigma-Aldrich; 0.04 mg/ml), creatine (Sigma-Aldrich; 1 mM), pyruvate (Sigma-Aldrich; 100 μg/ml), uridine (Sigma-Aldrich; 50 μg/ml), gentamycin (Life Technologies; 5 μg/ml) for 5 to 7 days.

To isolate pure human myoblasts, cultured cells were trypsinized and suspended in GM, and incubated with the following monoclonal anti-human antibodies, all obtained from BD Biosciences (Franklin Lakes, NJ, USA): anti CD56-AlexaFluor 488-, anti CD45 PE-CF594, anti CD144-PE, anti CD34-APC, anti CD146-PECy7. After a 30 min incubation at 4 °C, human myoblasts were washed and resuspended in GM. Human myoblasts, defined as CD56^+^CD146^+^CD45^−^CD34^−^CD144^−^, were sorted by flow cytometry using a FACS Aria (BD). Reanalysis was performed to check purity. Isotype control antibodies were PE-CF594-, APC-, PE-Cy7-, Alexa488 and PE-conjugated mouse IgG1 (all from BD Biosciences).

For flow cytometry analysis, myoblasts or MRC were incubated for 30 min on ice with appropriately diluted mouse monoclonal antibodies to human (all from BD Biosciences): CD56-Alexa488, CD146-PE-Cy7, CD73-FITC, CD90-FITC, CD105-FITC, Alkaline phosphatase-PerCP-Cy5.5, HLA ABC-PE, HLA DR-PE, CD82-PE and CD114-PE. Negative control samples received equivalent amounts of FITC-, Alexa488-, PerCPCy5.5, PE-Cy7- and PE labeled isotype-matched antibodies.

For cell cycle analysis, cells were permeabilized by incubation on ice for 20 minutes with Fix/Perm buffer (BD Biosciences). Cells were washed twice in Perm/Wash buffer (BD Biosciences) and incubated with a mouse anti human Ki67 AlexaFluor®647 or an isotype control AlexaFluor®647 mouse IgG1k for 30 minutes on ice (BD Biosciences). After washing with Perm/Wash buffer, cells were incubated for 15 minutes at 37 °C with 5 μl of Hoechst 33342 (1 mg/ml, Invitrogen). The fluorescence was measured using a LSR Fortessa (BD Biosciences) and data were analyzed using FlowJo 10.2 (FlowJo LLC, USA).

### Generation of human myogenic reserve cells

Human myoblasts were cultured in GM until confluence and then transferred to differentiation medium (DM). DM is a DMEM-based medium (Life Technologies) supplemented with bovine serum albumin (Sigma-Aldrich; 0.5 mg/ml), epidermal growth factor (Life Technologies; 10 ng/ml), insulin (Sigma-Aldrich; 0.01 mg/ml), creatine (Sigma-Aldrich; 1 mM), pyruvate (Sigma-Aldrich; 100 μg/ml), uridine (Sigma-Aldrich; 50 μg/ml), gentamycin (Life Technologies; 10 μg/ml). After 2 days in DM, both human myotubes and non-fused cells, defined as human MRC were observed. For transplantation experiments and flow cytometry analysis, human MRC were specifically isolated using a short trypsinization (30 seconds) that specifically removed all myotubes and left only quiescent undifferentiated cells adherent. To further eliminate small myotubes, trypsinized MRC were filtered using 20 μm pre-separation filters (Miltenyi Biotec, Bergisch Gladbach, Germany) before flow cytometry analysis or cell transplantation experiments. All *in vitro* and *in vivo* experiments were carried out with cells that had divided less than 20 folds.

### Western blot

Total protein extract was obtained by harvesting human myogenic cells (myoblasts, myotubes, MRC) after trypsinization in lysis buffer (50 mM Tris (pH 7.4), 250 mM NaCl, 5 mM EDTA, 50 mM NaF, 1 mM Na3VO4 et 1% Nonidet P40) containing protease inhibitors (Complete Tablet, Roche) and phosphatases inhibitors (PhosSTOP, Roche) on ice for 5 minutes. Cell lysates were centrifugated 5 min at 13’000 rpm and supernatant protein content was dosed using a Bradford assay.

Total proteins were separated on a 10% SDS- polyacrylamide gel and transferred to nitrocellulose membranes (Macherey-Nagel, Düren, Germany). Membranes were saturated in Tween/Tris-buffered saline (0.1% Tween 20, 20 mmol/l Tris–HCl (pH 7.5), and 137 mmol/l NaCl) containing 5% nonfat milk. Blots were incubated with the primary antibodies diluted in TTBS with 5% milk as follows: mouse anti myosin heavy chain (recognize all myosin heavy chain isoforms, MF20, 1/2000, Developmental Studies Hybridoma Bank, Iowa city, USA); mouse anti human Pax7 (1/300, Developmental Studies Hybridoma Bank), rabbit anti MyoD (1/500, Cell Signaling), rabbit anti Myf5 (1/200, Santa Cruz) and mouse anti α-tubulin (1/6000, Sigma). Blots were then incubated with horseradish peroxidase-conjugated goat anti-mouse or goat anti-rabbit antibodies (1/6000, BioRad, Hercules, CA). Blots were revealed using ECL reagents (Perkin-Elmer) and Hyperfilm MP (Amersham Biosciences, Piscataway, NJ). Image-J Software was used to quantify the level of protein expression.

### Renilla luciferase lentiviral vector

Virus was produced as described previously^38^. A three-plasmid expression system was used to generate second-generation lentiviral vectors by transient transfection of 293T cells. Infections of human myoblasts (10^5^ adherent cells) were performed at a multiplicity of infection (MOI) of 1, in the presence of polybrene (8 μg/ml) in GM. After overnight incubation, cells were washed and incubated in GM for 3 days. Transgene expression was confirmed by bioluminescence imaging before transplantation.

### Muscle laceration and intramuscular transplantation

Isoflurane (Abbott, Baar, Switzerland), supplemented with oxygen through a semi-closed circuit inhalation system, was used to anesthetize mice. Two days before cell transplantation, Gastrocnemius muscles were lacerated using a reliable method as previously described^60, 61^. Briefly, Gastrocnemius muscles were exposed and cut at 60% of the length from their distal insertion, through 75% of their width and 50% of their thickness. Muscles were then sutured using a polypropylen 6.0 (Ethicon, Somerville, NJ, USA). Thereafter, either human myoblasts or human MRC (5 × 10^5^ cells in 15 μl PBS) were injected into one location of the injured muscle, using a 25 μl syringe with a 29-gauge needle (Hamilton Company, Bonaduz, Switzerland). Mice received intraperitoneal injection of Temgesic (0.05 mg/kg, Essex Chemie, Luzern, Switzerland) for 3 days after muscle injury.

### Bioluminescence analysis

Imaging was performed using an IVIS 200 optical imaging system (PerkinElmer, Schwerzenbach, Switzerland) and data were processed using Living Image® Software (PerkinElmer, Schwerzenbach, Switzerland).

For live cell culture analysis, bioluminescence imaging (BLI) was performed immediately upon addition of the substrate (coelenterazine native; 1 μg/ml; Biotium, Hayward, CA, USA), diluted in cold PBS^++^ (containing Ca^2+^ and Mg^2+^). Acquisition time for *in vitro* experiments were 1 second. For *in vivo* experiments, bioluminescence was monitored immediately after Coelenterazine i.v injection in mice through the retro-orbital route (1 mg/kg diluted in 100 μl PBS^++^) and continuously for 12 minutes (4 consecutives acquisition of 3 min) A region of interest (ROI) was manually selected over the signal intensity. The area of the ROI was kept constant and signal recorded as maximum [photon/sec]. For technical reasons, the 3h-point post injection was defined as the reference 100% survival (day 0). BLI values measured at day 4, 7, 14 and 21 were related to the bioluminescence data obtained at day 0.

### Immunofluorescence

Myoblast cultures (in GM or DM) were fixed with 4% paraformaldehyde for 15 min at RT, washed twice in PBS and permeabilized with PBS containing 0.25% Triton X-100. After 3 washing steps in PBS, incubations with antibodies were undertaken in PBS adjusted to 2% Tween-10 and 2% goat serum. The following primary antibodies were used: mouse anti-pax7 antibody (1:150; DSHB, Iowa, USA), rabbit anti-MyoD antibody (1:500; Cell Signaling) and mouse anti-Ki67 antibody (1/250, BD). Secondary antibodies used for 1 h at room temperature (RT) were Alexa 488-labeled goat anti-rabbit IgG (1:1000; Molecular Probes, Eugene, OR) and Alexa 546-labeled goat anti-mouse IgG (1:1000; Molecular Probes). DAPI (100 ng/ml; Sigma) was used to visualize nuclei. Images were acquired using a Zeiss Axioskop 2 microscope. Ten random fields were acquired in each condition. Analysis to count Pax7 positive and/or MyoD positive labeled nuclei was done manually.

Differentiation state was quantified by assessing nuclear expression of the myogenic transcription factor MEF2 and by assessing expression of the myosin heavy chain protein. For this purpose, human cells were incubated with mouse anti-myosin heavy chain antibody (1:1000; DSHB, Iowa, USA) and with rabbit anti-MEF2 antibody (1:300, Santa Cruz Biotechnology Inc., Heidelberg, Germany). Fusion indexes were defined as the ratio of MEF2 positive nuclei count inside MF20 positive myotubes and divided by the number of total DAPI positive nuclei.

Immunofluorescence staining was also performed on cross-sectional 10 μm-thick muscle slices, obtained by the cryo-embedding in Tissue-Tek (Sakura, The Netherlands) of murine Gastrocnemius muscles injected with human cells. Cryosections were fixed in PBS-4% formaldehyde (Sigma-Aldrich) for 15 min, washed in PBS and unspecific sites blocked in PBS- 5% goat serum (Life Technologies) −0.3% Triton X100 (Sigma-Aldrich) for 1 hour at RT. Sections were stained with the following primary antibodies, overnight at 4 °C: mouse anti human lamin A/C (human specific, 1:100, Santa Cruz Biotechnology), rabbit anti lamin A/C (human specific, 1:2000, Abcam), rabbit anti dystrophin (1:500, Abcam), mouse anti Pax7 (1:20; DSHB, Iowa, USA), mouse anti spectrin (human specific, 1:50, LifeSpan BioScience, Seattle, WA, USA), rabbit anti-MyoD antibody (1:500; Cell Signaling) and rabbit anti laminin (1:1000, Abcam). After washing in PBS, slides were incubated 1 h at RT with AlexaFluor-conjugated secondary antibodies (1:1000, Molecular Probes). Nuclei were counterstained with DAPI (Sigma-Aldrich) and sections were mounted using polyvinyl alcohol mounting medium with DABCO (Sigma-Aldrich). Images were acquired using a Zeiss Axioskop 2 microscope.

### Sample size

Based on our preliminary data, a total sample size of 12 mice per group is needed with a standardized effect size of 1.2, a power analysis of 80% and p < 0.05.

### Statistical analysis

Statistical analyses were performed using unpaired Student’s t test with Bonferroni correction (α = 0.05) when necessary. Results were considered statistically significant when p < 0.05.
